# The Development of Robotic Technology in Cardiac and Vascular Interventions

**DOI:** 10.5041/RMMJ.10291

**Published:** 2017-07-31

**Authors:** Ali Pourdjabbar, Lawrence Ang, Ryan R. Reeves, Mitul P. Patel, Ehtisham Mahmud

**Affiliations:** Division of Cardiovascular Medicine, University of California, San Diego Sulpizio Cardiovascular Center, La Jolla, CA, USA

**Keywords:** Percutaneous coronary intervention, peripheral vascular intervention, robotically assisted PCI

## Abstract

Robotic technology has been used in cardiovascular medicine for over a decade, and over that period its use has been expanded to interventional cardiology and percutaneous coronary and peripheral vascular interventions. The safety and feasibility of robotically assisted interventions has been demonstrated in multiple studies ranging from simple to complex coronary lesions, and in the treatment of iliofemoral and infrapopliteal disease. These studies have shown a reduction in operator exposure to harmful ionizing radiation, and the use of robotics has the intuitive benefit of alleviating the occupational hazard of operator orthopedic injuries. In addition to the interventional operator benefits, robotically assisted intervention has the potential to also be beneficial for patients by allowing more accurate lesion length measurement, stent placement, and patient radiation exposure; however, more investigation is required to elucidate these benefits fully.

## INTRODUCTION

Robotic technology has been used in medicine for several decades, primarily in the fields of surgery^1^–^3^ and radiation therapy.^4^,^5^ The use of robotics in cardiovascular medicine began in the early 2000s, and since its introduction robotics has made a tremendous impact in the field. Today robotic technology is commonly used to assist in a number of surgical procedures including minimally invasive atrial septal defect closure, mitral valve repair, coronary artery bypass graft surgery, and, more recently, in electrophysiology procedures involving radiofrequency arrhythmia ablation.^6^–^9^

Despite significant advances in pharmacotherapy and interventional device technology, the performance of percutaneous coronary and peripheral vascular interventional procedures has remained relatively unchanged over the past four decades. As a result, significant occupational hazards exist for interventional operators, who are exposed to the risk of both orthopedic and radiation-related complications. The emergence of robotic technology within the field of interventional cardiology provides cardiologists with a novel, safe, and effective tool to provide remote care for patients while reducing the risk of long-term operator harm. Currently, it is approved by the United States Food and Drug Administration (FDA) for both coronary and peripheral vascular interventions.^10^ In this review we outline the detrimental long-term hazards facing interventional operators, while highlighting the available clinical evidence behind the emergence of robotics in the field.

## ROBOTICS IN INTERVENTIONAL CARDIOLOGY

A significant driving force behind the emergence of robotics in interventional cardiology is the growing evidence highlighting occupational hazards associated with the field.^11^ These hazards include orthopedic injuries related to the long-term use of heavy lead aprons during percutaneous interventional procedures. A recent survey by Klein et al.^12^ reports that the prevalence of orthopedic injuries including cervical and lumbar disc disease might be as high as 42% among invasive cardiologists, a finding that was directly related to the years of practice after fellowship.

Even more concerning are the long-term consequences of chronic exposure to ionizing radiation. These complications include increased risk of cataract development^13^,^14^ and a possible association with increased risk of malignancy including head and neck tumors.^15^,^16^ Recent reports have also shown a significantly higher proportion of left-sided tumors among interventionalists potentially due to the close proximity of the left side of the head to the radiation source.^17^ In the recently published BRAIN study, we reported that radiation exposure to invasive cardiologists was significantly higher on the left and center as compared to the right side of the cranium.^18^ Although a direct link between operator radiation exposure and brain tumors, and more specifically left-sided tumors, has not been established, these reports do raise concerns and highlight potential associations. Radiation safety is a very important aspect of the practice of interventional cardiology, and a number of safety precautions including collimation, use of Eco Dose Fluoroscopy,^19^ and operator education^20^ have significantly reduced radiation exposure. Despite these efforts, the long-term risk of complications associated with chronic radiation exposure can never be completely eliminated. Robotic technology remotely enabling percutaneous coronary intervention (PCI) reduces the orthopedic risk by eliminating the need for wearing the heavy lead apron, and also significantly reduces operator radiation exposure by 95%–97%.^21^,^22^

In addition to the occupational hazard reduction for the operator, using the robotic platform, operators can make precise lesion length measurements, allowing for the selection of appropriate-length stents.^23^ As a result, potentially a lower number of stents are utilized, and, by reducing longitudinal geographic mismatch, an improvement in longer-term revascularization rates might be seen.^24^

## CORPATH 200 ROBOTIC SYSTEM

**Figure 1:**
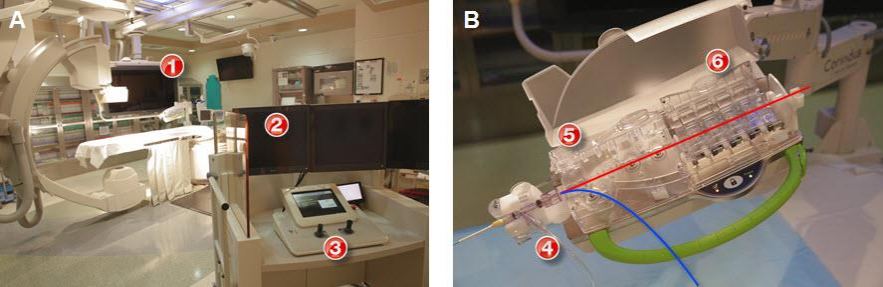
The CorPath 200 Vascular Robotic System. **(A)** Robotic PCI platform—robotic console and tableside drive (CorPath 200, Corindus, Waltham, MA) and robotic cassette. ➀ Robotic arm and cassette; ➁ interventional cockpit; ➂ control console. **(B)** Robotic cassette demonstrating the placement for the guidewire (red) and the balloon/stent (blue). ➃ Driver for rapid-exchange catheters; ➄ driver for 0.014” guidewires; ➅ mechanical torque system for 0.014” guidewires.

The clinical efficacy of the prototype robotic PCI system was first reported in 2006 by Beyar et al. with clinical and technical success rates of 100% and 93%, respectively.^25^ This experimental remotely guided PCI system was refined and introduced as the CorPath 200 (Corindus, Waltham, MA, USA) vascular robotic system in 2012 (Figure 1, left panel). The system consists of an interventional cockpit and a robotic arm mounted on the catheterization bedside rail. This robotic arm contains a drive housing a single-use sterile cassette (Figure 1, right panel), which is connected to the guiding catheter after manually engaging the coronary artery. The currently available system is compatible with all 0.014-inch coronary guide wires and standard rapid exchange balloon and stent delivery systems. The interventional cockpit is located within the cardiac catheterization laboratory and is connected via cables to the bedside drive. It contains monitors that display the live fluoroscopic image and hemodynamic data. The robotic system enables the operator to advance and retract rapid exchange balloons and stents remotely. Additionally, the operator can rotate and advance the guide wire transmitting torque and permitting guide wire manipulation. Passive control of the guiding catheter is possible with guide wire and balloon manipulation. The next generation CorPath GRX system that has recently received FDA approval contains active guide control, allowing the operator to control the guide catheter remotely. However, at this point there are no clinical data available with this new-generation system.

## CORPATH 200 AND CORONARY INTERVENTIONS

After the first-in-human feasibility study using robotic PCI,^21^ the PRECISE trial was initiated. This study enrolled 164 patients with *de novo* coronary stenosis of at least 50%,^22^ with the primary objective of evaluating the safety and the clinical and technical performance of the CorPath 200 system in the delivery and manipulation of coronary guide wires, balloons, and stents during PCI. The two end-points were: (1) clinical procedural success, defined as <30% residual stenosis post-robotic PCI as defined by quantitative coronary analysis (QCA) in the absence of any major adverse cardiovascular event (MACE); and (2) technical success, defined as successful advancement and retraction of the PCI devices (guide wire, balloons, and stents) via the robotic system without conversion to manual operation. Secondary end-points included in-hospital and 30-day MACE, and operator radiation exposure. The majority of lesions treated in this study were American College of Cardiology/American Heart Association (ACC/AHA) types A and B (>87%) with an average lesion length of 12.2±4.8 mm. Clinical and technical success rates were 97.6% and 98.8%, respectively, with a >95% reduction in median operator radiation exposure reported (Table 1). Based on the results of this study, the FDA approved the CorPath 200 system for PCI in 2012. In 2015, based on additional studies, this approval was extended to radial access PCI.^26^,^27^

**Table 1:** Study Design, Patient Characteristics, and Outcomes of the Major Studies Investigating Robotic Interventions.

	Granada et al.	PRECISE Trial	CORA PCI	RAPID
Author (reference)	**Granada et al.**^21^	**Weisz et al.**^22^	**Mahmud et al.**^30^	**Mahmud et al.**^38^
Study design	Prospective, single-arm, single-center, open-label, non-randomized	Prospective, single-arm, multi-center, open-label, non-randomized	Prospective, comparative, single-center, open-label, non-randomized	Prospective, single-arm, single-center, open-label, non-randomized
Lesion Location	Coronary	Coronary	Coronary	Peripheral
*n*	8	164	108	20
Lesions, *n*	8	164	157	29
Clinical success, %	100	97.6	98.8	100
Technical success, %	97.9	98.8	91.7	100
Type A/B1 lesions, *n* (%)	8 (100)	112 (68)	35 (22)	–
Type B2/C lesions, *n* (%)	0 (0)	52 (32)	122 (78)	–
In-hospital MACE^*^, *n* (%)	0 (0)	4 (4.2)	6 (5.6)	0 (0)
Radiation reduction (operator), %	97	95.2	–	–
Lesion length, mm	11.4	12.2±4.8	22.2±10.6	33.1±15.5
Mean diameter stenosis, %	63.1±15	64.1±10.9	84.9±9.2	85.5±11.0

* Using universal definition of myocardial infarction.

**Figure 2:**
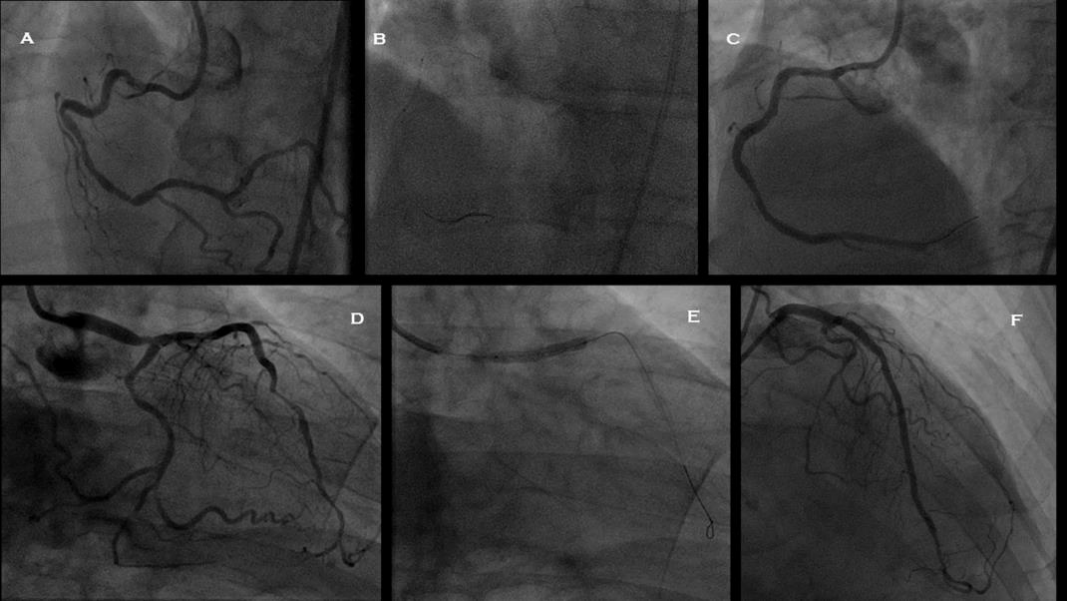
Exemplary Procedures of Complex Robotic PCI Showing Pre- and Post-PCI Angiograms in a Patient with Multi-vessel Disease. **(A)** Angiogram of the right coronary artery (RCA) demonstrating a tortuous artery with severe obstructive lesions in the mid and distal segments. **(B)** Demonstrates the use of a buddy wire and a Wiggle wire, both advanced robotically for the delivery of a stent to the mid and distal RCA. **(C)** Final angiogram after stenting of the mid and distal RCA. **(D)** Right anterior oblique (RAO) caudal view of the left coronary system demonstrating severe distal left anterior descending (LAD) and distal left main lesions. **(E)** Stent delivery and deployment within the distal left main into the proximal LAD. **(F)** Final angiogram of the left coronary artery after stenting of the distal left main into the LAD and the distal LAD.

The majority of lesions treated in the PRECISE study were simple, relatively short-length lesions, with only a small proportion of type C lesions (12.8%). Since then several reports have demonstrated the feasibility of robotic PCI for complex procedures including multi-lesion coronary artery disease, allograft vasculopathy, vein graft PCI, unprotected left main PCI, and ST elevation myocardial infarction (STEMI).^28^,^29^ The Complex Robotically Assisted (CORA) PCI study evaluated the safety and feasibility of robotically assisted PCI and compared clinical outcomes against manual PCI in an all-comers, real-world clinical practice.^30^ This study enrolled 334 patients, with 108 patients (157 lesions) in the robotic group and 226 patients (336 lesions) in the manual group. The majority of treated lesions were classified as ACC/AHA B2/C (78% robotic; 69% manual). Robotic technical success was reported in 91.7%, with manual conversion required in 7.4% of procedures. Comparable clinical success (robotic 99.1% versus manual 99.1%, *P*=1.0) was observed in both the robotic and manually treated patients (Table 1). No difference was observed in stent utilization, contrast utilization, or patient radiation exposure (dose area product [DAP] or total fluoroscopy time). However, the use of robotics was associated with longer total procedure time (44:30±26:04 versus 36:34±23:03 min:sec, robotic versus manual PCI, respectively; *P*=0.005). Further analysis revealed that the increase in procedure time was only observed in relatively simpler lesions while no difference in procedure time existed as the treated lesion complexity increased (Figure 2). An analysis of the extremely complex cohort from the CORA PCI study also reported comparable safety and efficacy of the robotic and manual approaches.^31^ The rate for conversion from robotic to manual PCI was low, occurring in 13% of patients, with no difference observed in the utilization of stents, number of lesions treated, total procedure or fluoroscopy time, contrast use, or dose area product.

Thus far, the available clinical data for robotic PCI are based on the CorPath 200 platform, which is set up with the robotic arm and operator cockpit being in the same room as the patient. However, a new emerging concept in the field of robotic PCI is remote robotic interventions or “telestenting” where the interventional cardiologist can perform the procedure using a remotely placed operator cockpit in a room removed from the catheterization suite. The feasibility of “telestenting” was recently reported by Madder et al., in a single-center prospective observational study, enrolling 20 patients with obstructive coronary disease.^32^ In this study, the robotic cockpit was located in an isolated room away from the main interventional suite, with telecommunication devices enabling audio and video communication between the operating physician and the rest of the catheterization laboratory team. Technical success and procedural success was 86.4% and 95%, respectively. There were no major adverse events (mortality or repeat revascularizations prior to hospital discharge) in the study cohort. This study demonstrates the feasibility of remote robotic PCI, but more importantly highlights one of the major potential benefits offered by this technology. Larger studies are required to confirm its feasibility and also to determine if future advancement in robotic technology could facilitate telestenting over longer geographic distances. One could surmise that with telestenting, PCI could be performed in remote locations or a single highly experienced operator could remotely assist lower-volume less experienced operators.

## CORPATH 200 AND PERIPHERAL VASCULAR INTERVENTIONS

Although the feasibility of robotic peripheral vascular intervention (PVI) was previously reported with the Hansen Magellan robotic system (Hansen Medical, Mountain View, CA, USA),^33^–^36^ for various reasons this system has never gained traction for use in clinical practice. As PVI procedures are performed tableside similar to PCI, similar operator radiation and orthopedic risks exist. In fact, as compared to PCI, peripheral vascular interventional procedures are associated with higher radiation exposure for operators.^37^ Therefore, the potential for operator benefit with the use of robotic technology also exists during PVI.

We performed the RAPID (Robotic-Assisted Peripheral Interventions for Peripheral Artery Disease [PAD]) trial,^38^ a study to evaluate the feasibility of the CorPath 200 robotic system in the management of PVI. Patients with symptomatic PAD due to either lifestyle-limiting claudication or critical limb ischemia affecting the femoropopliteal vessels and requiring PVI were prospectively enrolled. A total of 20 patients with 29 lesions were enrolled in this pilot study, with 89.7% of the lesions involving the superficial femoral artery and 10.3% involving the popliteal artery. The study end-points were device technical success and clinical success. Technical success was defined as cannulation of the target vessel with the guide wire and dilating angioplasty balloon, while clinical procedural success was defined as <50% residual stenosis without an unplanned switch to a manual procedure or device-related serious adverse event in the peri-procedural period. The investigators reported 100% technical and clinical success, without any significant adverse events. In 2016, the findings of the RAPID study resulted in FDA approval of the system for clinical use in PVI and led to the design of the RAPID II study. This study is currently enrolling patients with symptomatic femoropopliteal disease and is examining the system’s ability to deliver drug-coated balloons and stents; it will also be reporting 30-day outcomes.

**Figure 3:**
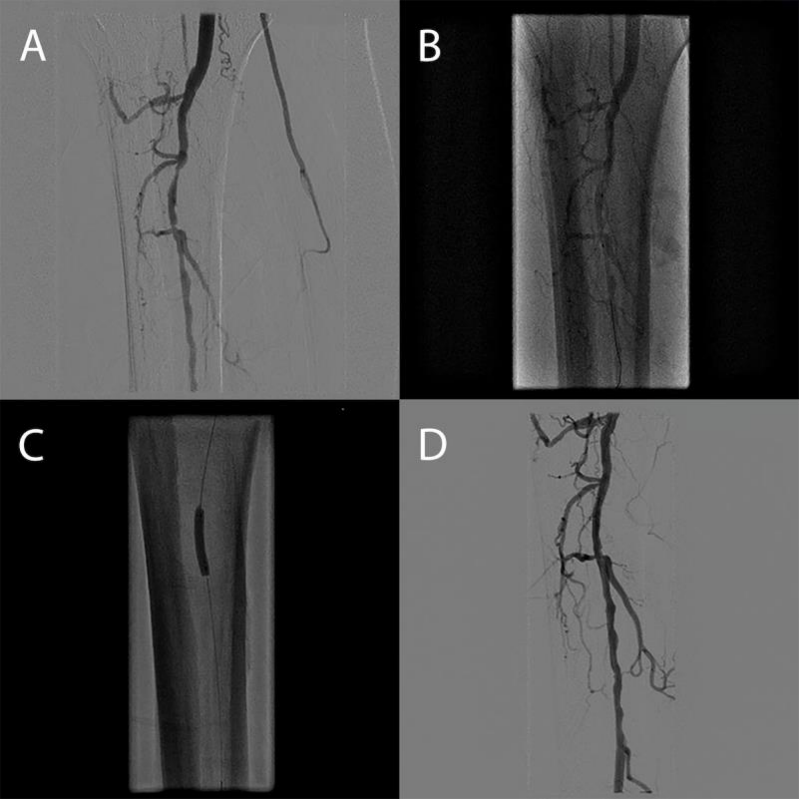
Right Lower Extremity below the Knee Robotic Revascularization. **(A)** Preprocedural angiogram demonstrating a focal stenosis within the tibioperoneal (TP) trunk, a focal lesion in the proximal peroneal artery, and occluded anterior and posterior tibialis arteries. **(B)** Follow-up angiography after robotically assisted balloon angioplasty of the peroneal and TP trunk (3.0×20 mm Maverick RX balloon, Boston Scientific, Marlborough, MA, USA) revealing elastic recoil. **(C)** Additional balloon angioplasty of both target lesions (3.5×20 mm Maverick RX balloon, Boston Scientific, Marlborough, MA, USA). **(D)** Final angiogram demonstrating <30% residual stenosis and no flow-limiting dissections. From Behnamfar et al. J Invasive Cardiol 2016 Nov; 28(11): E128–E131, used with permission.

Infrapopliteal lesions account for a significant number of PAD treated percutaneously.^39^ Furthermore these lesions are ideal targets for robotic intervention with the currently available system. The size of the target infrapopliteal vessels typically requires the use of coronary interventional equipment including balloons and 0.014-inch guide wires, which are compatible with the current CorPath 200 system, and they are often treated with balloon angioplasty alone. Behnamfar et al. recently reported the first case of successful percutaneous management of below-the-knee PAD using robotically assisted balloon angioplasty with the CorPath 200 system.^40^ Successful balloon angioplasty was performed using this system for a 56-year-old man with a focal stenosis in the tibioperoneal trunk and proximal peroneal artery. The procedure was performed without any complications, demonstrating that robotic PVI can be performed for below-the-knee disease (Figure 3). However, further clinical data are required fully to examine the safety and effectiveness of this technique in a larger sample of patients with below-the-knee disease.

## LIMITATIONS AND FUTURE DIRECTIONS

While robotic PCI continues to show great promise, a number of limitations and hurdles remain. Although the currently available evidence demonstrates the safety and feasibility of robotic PCI for both simple and complex lesions, its utility in certain complex lesions/scenarios—including STEMI, bifurcation disease requiring advanced stenting techniques, severely calcified lesions requiring atherectomy, and chronic total occlusions—remains unclear. Although small series and case reports have shown encouraging results,^41^ larger studies and iterative improvement in technology are required prior to robotic PCI being used routinely in these lesions. Improved precision and accuracy with the robotic system to measure lesion length has not translated into a difference in resource utilization.

Finally, the current CorPath 200 system has only been validated with a 0.014-inch guide wire and rapid exchange balloon and stent systems. Further improvements in the robotic platform and system including compatibility with 0.018 and 0.035-inch guide wires, over-the-wire balloon catheters, drug-coated balloons, intravascular imaging catheters, and atherectomy devices will broaden its application. The development of active guide catheter control in the CorPath GRX system has been an eagerly awaited step and should further enhance the use of this technology for the management of patients with complex coronary disease.

Another exciting development in the field of robotics and interventional cardiology is the potential use of this technology in structural heart disease, specifically transcatheter aortic valve replacement (TAVR) procedures. Early studies using a silicone *in vitro* model of the aorta, aortic arch, and stenotic valve with a TAVR simulation program have reported improved ability to navigate through the aorta with significant reduction in equipment contact with the aortic wall which could have an impact on embolic cerebrovascular events during the delivery of large equipment and sheaths.^42^,^43^ Robotic TAVR and mitral valve repair represent yet another exciting aspect of interventional cardiology where robotics is emerging as a useful tool; however, the applicability of this technology requires clinical validation.

## CONCLUSIONS

The development and refinement of robotic systems represents the dawn of a new era in the field of interventional cardiology. In the vast majority of patients treated in clinical practice, robotically assisted PCI is safe and results in clinical outcomes comparable to those using a manual approach. The feasibility of robotic PVI has also been demonstrated. Hence, robotic technology offers a potential solution to the occupational hazards facing interventional physicians by limiting radiation exposure and reducing orthopedic strain. In the future, robotics has the potential to expand the availability of PCI to remote areas with telestenting and to address structural heart disease interventions.

## Abbreviations

ACC/AHA: American College of Cardiology/American Heart Association
CORA: complex robotically assisted
FDA: United States Food and Drug Administration
MACE: major adverse cardiovascular event
PAD: peripheral artery disease
PCI: percutaneous coronary intervention
PVI: peripheral vascular intervention
STEMI: ST elevation myocardial infarction
TAVR: transcatheter aortic valve replacement.
